# Understanding the Digital Gap Among US Adults With Disability: Cross-Sectional Analysis of the Health Information National Trends Survey 2013

**DOI:** 10.2196/rehab.8783

**Published:** 2018-03-16

**Authors:** Eun Ji Kim, Yiyang Yuan, Jane Liebschutz, Howard Cabral, Lewis Kazis

**Affiliations:** ^1^ Donald and Barbara Zucker School of Medicine at Hofstra/Northwell Manhasset, NY United States; ^2^ University of Massachusetts Medical School Worcester, MA United States; ^3^ University of Pittsburgh Pittsburgh, PA United States; ^4^ Boston University School of Public Health Boston, MA United States

**Keywords:** disability, health information, Internet, health care provider, trust, psychosocial factors

## Abstract

**Background:**

Disabilities affect more than 1 in 5 US adults, and those with disabilities face multiple barriers in accessing health care. A digital gap, defined as the disparity caused by differences in the ability to use advanced technologies, is assumed to be prevalent among individuals with disabilities.

**Objective:**

This study examined the associations between disability and use of information technology (IT) in obtaining health information and between trust factors and IT use. We hypothesized that compared to US adults without disabilities, those with disabilities are less likely to refer to the internet for health information, more likely to refer to a health care provider to obtain health information, and less likely to use IT to exchange medical information with a provider. Additionally, we hypothesized that trust factors, such as trust toward health information source and willingness to exchange health information, are associated with IT use.

**Methods:**

The primary database was the 2013 Health Information National Trends Survey 4 Cycle 3 (N=3185). Disability status, the primary study covariate, was based on 6 questions that encompassed a wide spectrum of conditions, including impairments in mobility, cognition, independent living, vision, hearing, and self-care. Study covariates included sociodemographic factors, respondents’ trust toward the internet and provider as information sources, and willingness to exchange medical information via IT with providers. Study outcomes were the use of the internet as the primary health information source, use of health care providers as the primary health information source, and use of IT to exchange medical information with providers. We conducted multivariate logistic regressions to examine the association between disability and study outcomes controlling for study covariates. Multiple imputations with fully conditional specification were used to impute missing values.

**Results:**

We found presence of any disability was associated with decreased odds (adjusted odds ratio [AOR] 0.65, 95% CI 0.43-0.98) of obtaining health information from the internet, in particular for those with vision disability (AOR 0.27, 95% CI 0.11-0.65) and those with mobility disability (AOR 0.51, 95% CI 0.30-0.88). Compared to those without disabilities, those with disabilities were significantly more likely to consult a health care provider for health information in both actual (OR 2.21, 95% CI 1.54-3.18) and hypothetical situations (OR 1.80, 95% CI 1.24-2.60). Trust toward health information from the internet (AOR 3.62, 95% CI 2.07-6.33), and willingness to exchange via IT medical information with a provider (AOR 1.88, 95% CI 1.57-2.24) were significant predictors for seeking and exchanging such information, respectively.

**Conclusions:**

A potential digital gap may exist among US adults with disabilities in terms of their recent use of the internet for health information. Trust toward health information sources and willingness play an important role in people’s engagement in use of the internet for health information. Future studies should focus on addressing trust factors associated with IT use and developing tools to improve access to care for those with disabilities.

## Introduction

Disability is a common condition in the United States [1]. According to recent data from the Behavioral Risk Factor Surveillance System (BRFSS 2013), more than 1 in 5 adults (22.2%) reported having disability [1]. Risk of disability increases with age, and the number of individuals with disabilities will likely rise as the elderly population continues to grow. Those with disabilities have been shown in previous studies to have a higher demand for health information but often experience a lack of such information compared to those without disabilities [2].

The internet has the potential to bridge disparities in obtaining health information. Americans widely use the internet to obtain health information [3,4]. Internet use, in terms of obtaining health information and exchanging medical information, has shown to improve health outcomes and lower health care costs [5-11]. Rapid advancements in information technology (IT) and increasing ownership of mobile devices make electronic health information more easily accessible. For those with physical and sensory impairments, the internet and IT have created potential opportunities to offer health information that can be accessed by those with disabilities [3,12-18].

Despite these advancements, those with disabilities experience a digital gap, defined as a disparity caused by differences in the ability to use advanced technologies [19]. The gap is partially explained by the higher proportion of people with disabilities having characteristics associated with lower use of the internet to obtain health information compared to people without disabilities; they tend to be older in age, unemployed, less educated, and have low income [20-28]. Additionally, studies show an association between psychosocial factors associated with the digital gap [4,29]. However, there is a lack of research examining an association between trust factors, specifically trust toward a health information source and willingness to exchange medical information, associated with the digital gap among people with disabilities at a national level.

For this study, we used recent data from the National Cancer Institute’s Health Information National Trends Survey (HINTS) to explore the association between (1) disability and use of the internet as the primary information source and (2) disability and IT to exchange medical information with a health care provider. Next, we examined the association by specific disability conditions and then studied trust factors associated with the internet and IT use. We hypothesized that US adults with disabilities are less likely to use the internet for health information, more likely to use a health care provider for health information, and less likely to use IT to exchange medical information with a provider compared to those without disabilities. We also hypothesize that trust factors such as trust toward a health information source and willingness to exchange health information with a health care provider are associated with the utilization.

## Methods

### Study Sample

We used data from HINTS 4, Cycle 3 (2013) [30]. HINTS is a nationally representative mail survey that contains questions about health information seeking behaviors and health information sources. The sample design for this survey consisted of 2 stages: a stratified sample of household addresses was first selected from a residential file, and then 1 adult in the household was identified to complete the survey. The survey was mailed in 2 versions (English and Spanish), with the majority of the responses collected from the English version (94.7%) [30]. The unweighted sample size for HINTS 4 Cycle 3 was 3185. Due to the skip pattern in the survey instrument, the study sample for the first 2 hypotheses only included those who had ever sought health-related information (N=2508). For the third hypothesis, all respondents were included in the analyses (N=3185).

### Main Outcome

Main outcomes of this study were defined by 3 questions: “The most recent time you looked for information about health or medical topics, where did you go first?” (HINTS A2); “Imagine that you had a strong need to get information about health or medical topics. Where would you go first?” (HINTS A8); and “In the past 12 months, have you used any of the following to exchange medical information with a health care professional?” (HINTS B6).

Specifically, hypotheses 1 and 2 examined sources of health information reported by respondents. In the survey, a list of common health information sources was provided, including health care provider, internet, family, etc. These sources were exclusive and respondents were asked to identify only one. For the purpose of this study, we focused on internet and health care provider. In addition, the survey also differentiated actual use, defined as the primary health information source that they had used recently (HINTS A2), and hypothetical use, defined as the source that they would use to obtain health information when there is a strong need for such information (HINTS A8). In this study, we examined both actual use and hypothetical use of internet and health care provider as health information sources.

Hypothesis 3 looked at exchanging health information via IT. Respondents of the survey were asked to identify the routes they had used to exchange medical information with their health care providers (HINTS B6). We defined IT use as exchanging medical information with health care professionals via any of the following: email, text message, app on a smartphone or mobile device, video conference, or social media.

### Disability Measure

The survey included questions recommended by the US Department of Health and Human Services to measure disability in 6 domains: hearing (deaf or serious difficulty in hearing), vision (blind or serious difficulty in seeing even with glasses), cognition (serious difficulty concentrating, remembering, or making decisions because of a physical, mental, or emotional condition), mobility (serious difficulty walking or climbing stairs), self-care (difficulty dressing or bathing), and independent living (difficulty doing errands alone because of a physical, mental, or emotional condition) [31]. This classification aligns with the comprehensive measures for defining disabilities in the World Health Organization’s International Classification of Function, Disability, and Health [32] and emphasizes the impact of the disabilities on functional limitations. Having one or any combination of these conditions was classified as “any disability.”

### Trust Factors

We evaluated participants’ trust toward various health information sources for hypotheses 1 and 2 and willingness to exchange medical information with providers for hypothesis 3. We dichotomized participants’ trust toward getting health information from the internet, health care providers, family, or friends to “a lot” versus “some/little/not at all,” which respectively captured higher and lower levels of trust [33]. For the willingness to electronically exchange medical information, we assigned a score of 1 (“not at all”) to 4 (“a lot”) for each category of medical information and calculated the average willingness score for each respondent.

### Covariates

We also controlled for several factors known for their association with use of health information sources: gender, age, marital status, race/ethnicity, education, insurance status, annual income level, designated regular provider, mobile device ownership, self-rated health status, respondents’ perceptions of the importance of the patient accessing medical information electronically, and existence of an electronic medical record system [34]. The response to the question, “Overall, how confident are you that you could get advice or information about health or medical topics if you needed it?” (HINTS A6), was used as a proxy for health literacy with respect to the ability to obtain health information [35].

### Statistical Analysis

We used multiple imputation with fully conditional specification to impute all variables with missing values in our statistical model. The imputation model for race/ethnicity included strong predictors of this variable: survey language (English or Spanish), Hispanic household stratum, birthplace (United States or foreign born), income level, and English proficiency. For other variables, imputation models included all variables in this study. Our implementation of the fully conditional specification approach incorporated sample weights and design effects in the imputation to account for the complex sample design [36]. We generated 10 imputed datasets for subsequent analysis. The results of all of our analytic models were computed using standard methods for combining model estimates and standard errors across the multiple imputed data sets.

We assessed sample characteristics of the entire study sample and by disability status. Unweighted frequencies and weighted percentages were presented. We examined the difference between any disability and no disability for each sample characteristic using chi-square tests with Rao-Scott correction for categorical variables and *t* tests for continuous variables and reported the corresponding *P* values.

We conducted logistic regressions to examine the association between each sample characteristic with the outcomes. We evaluated 2-way interactions between disability status and sample characteristics. Because none of these 2-way interactions were statistically significant, we only included main effects in the final model. To examine our hypotheses, we performed multivariable logistic regression adjusting for relevant covariates, and as noted above, compiled the results across imputed datasets. We reported the range of *c*-statistics from the 10 models with imputed values for each outcome to evaluate the goodness of fit. Furthermore, to examine the potential heterogeneity within the disability group created by its composite measure, we conducted multivariable logistic regression for each of the 6 disability subgroups controlling for the same covariates. The reference group for each disability type comprised those without any type of disability. Data were weighted using jackknife variance estimation with 50 replicate weights to produce a sample representative of 235 million US adults [30].

Statistical analyses were conducted in SAS 9.4 (SAS Institute Inc). The Boston University Institutional Review Board approved this study.

## Results

### Sample Characteristics

Of the total sample, 19.6% (796/3185) reported any type of disability (Table 1). Approximately two-thirds (473/796, 66.7%) of those with disability rated their general health as excellent, very good, or good, which was significantly lower than those without disability (2086/2274, 90.9%). Among those with disabilities, difficulty with cognition was the most prevalent (310/796, 49.1%), followed by mobility (459/796, 48.6%) and hearing (242/796, 29.9%) (Multimedia Appendix 1). Compared to the no-disability group, those with disabilities had a significantly higher proportion (with disability, 351/796, 34.3%; without disability, 523/2274, 12.6%) of individuals aged 65 years or older (*P*<.001). Higher proportions of people with disabilities were single (*P*<.001) and had low household income (*P*<.001), low health literacy (*P*=.02), and low levels of education (*P*<.001) compared to those without disabilities. Overall, more than half of the sample (1794/3185, 55.5%) had high confidence in obtaining health information, a proxy for higher health literacy.

Next, we examined characteristics associated with electronic health information communication between those with and without disability (Table 2). Overall, more than one-third (986/3185, 36.4%) of the study participants reported not having a regular health care provider; the majority (2721/3185, 87.7%) of respondents reported their providers maintained health records in a computerized system. Results suggest the use of an electronic medical record system by the health care providers to exchange medical information was independent of disability status (with disability, 697/796, 89.0%; without disability, 2024/2274, 87.2%; *P*=.47). The majority of the study sample (2754/3185, 91.7%) owned 1 or multiple electronic mobile devices (with disability, 622/796, 82.5% without disability, 2132/2274, 94.1%; *P*<.001). A smaller percentage of those with disabilities rated the high importance of electronically accessing their own medical information (with disability, 454/796, 58.2%; with disability, 1493/2274, 66.5%, *P*<.001).

### Seeking Health Information

In the study sample, 69.4% (1334/2508) reported using the internet in their most recent search for health information; a smaller percentage (969/2508, 46.6%) of respondents reported referring to the internet as their first choice when there was a strong need for health information. In bivariate analysis, those with disabilities, compared to those without disabilities, were significantly less likely to use the internet as the source for health information in both actual (odds ratio [OR] 0.32, 95% CI 0.24-0.45) and hypothetical situations (OR 0.42, 95% CI 0.29-0.60). Only 13.6% (362/2508) of all respondents reported high trust toward the health information from the internet, and there was no difference in trust between those with and without disabilities (*P*=.54).

Adjusting for other covariates, those with disabilities remained significantly less likely to use the internet in their most recent search for health information (adjusted OR [AOR] 0.65, 95% CI 0.43-0.98) but not in their proposed hypothetical use (AOR 0.66, 95% CI 0.41-1.06). Those with a higher level of trust toward the internet were 3.62 times and 2.53 times more likely to use it in actual and hypothetical searches for health information, respectively, compared to respondents with lower trust (Table 3).

Subgroup analyses were performed to identify specific disability conditions associated with lower internet use in the most recent search for health information (Table 4). Compared to those without any disability and controlling for other covariates, those with impairment in vision (AOR 0.27, 95% CI 0.11-0.65) and mobility (AOR 0.51, 95% CI 0.30-0.88) were less likely to have used the internet in their last search for health information.

### Seeking Health Information From a Health Care Provider

The majority of the respondents (1733/2508, 70.1%) indicated a high level of trust for a health care provider as their primary information source. When respondents have a strong need for health information, 45.1% (1173/2508) responded that they would turn to a doctor or health care provider, which was nearly 3-fold the proportion of those who reported actually using a health care provider as the information source (408/2508, 14.8%). Compared to those without disabilities, those with disabilities were significantly more likely to consult a health care provider for health information, in both actual (OR 2.21, 95% CI 1.54-3.18) and hypothetical situations (OR 1.80, 95% CI 1.24-2.60). This significant association shown in the bivariate analysis between disability status and seeking health information from a provider was no longer statistically significant when controlling for other covariates (Table 3).

High trust for providers was a significant factor in seeking health information from a provider when the patient had strong needs; those who had high trust toward a provider (AOR 2.26, 95% CI 1.71-2.99) were more likely to seek health information from a provider. On the other hand, those with higher trust for health information online were significantly less likely to use a provider as either the actual (AOR 0.38, 95% CI 0.19-0.78) or hypothetical source (AOR 0.47, 95% CI 0.32-0.69) of health information compared to respondents with a lower level of trust, when adjusting for other covariates.

### Exchanging Health Information via Information Technology With a Health Care Provider

Those without disabilities indicated they were statistically significantly more willing to exchange health information via IT (mean willingness score 2.62, SD 0.03) compared to those with disabilities (mean willingness score 2.39, SD 0.07; *P*<.01) (Table 2). Willingness to share health information (AOR 1.88, 95% CI 1.57-2.24) was significantly associated with exchanging medical information with a health care provider via IT. Contrary to our hypothesis, there was no association between medical information exchange via IT and the disability condition (OR 0.76, 95% CI 0.53-1.09; AOR 1.37, 95% CI 0.92-2.04). This finding persisted in analyses with all 6 disability conditions. For those with disability in vision, they were shown to have marginally significant higher likelihood (AOR 1.55, 95% CI 0.99-2.43) of exchanging medical information via IT with health care providers (Table 4).

**Table 1:** Sociodemographic characteristics of the study sample.

Characteristic		Total (n=3185), %^a^	Any disability (n=796), %^a^	No disability (n=2274), %^a^	*P* value^b^
**General health**					
	Excellent, very good, or good	86.1	66.7	90.9	<.001
	Fair or poor	13.9	33.3	9.1	—
**Gender**					
	Male	48.4	46.3	49.2	.43
	Female	51.6	53.7	50.9	—
**Age group, years**					
	Younger than 65	82.8	65.7	87.4	<.001
	65 or older	17.2	34.3	12.6	—
**Marital status**					
	Married or living as married	58.8	47.0	61.8	<.001
	Single^c^	41.2	53.0	38.2	—
**Race/ethnicity**					
	Non-Hispanic white	66.9	63.7	68.1	<.001
	Hispanic	15.4	15.7	14.7	—
	Non-Hispanic black or African American	10.5	16.4	9.3	—
	Non-Hispanic Asian	5.1	2.7	5.6	—
	Other	2.1	1.5	2.3	—
**Education**					
	Less or complete high school	34.1	50.0	29.4	<.001
	Some college	32.7	33.0	32.8	—
	College graduate	33.2	17.0	37.7	—
**Health literacy**					
	High	55.5	49.8	57.3	0.02
	Low	44.5	50.2	42.7	—
**Insurance**					
	Have insurance	83.0	83.2	82.6	—
	No insurance	17.0	16.8	17.4	0.85
**Income^d^**					
	Less than $20,000	20.9	40.9	15.9	<.001
	$20,000 to $34,999	14.3	16.4	13.8	—
	$35,000 to $49,999	14.6	14.5	14.6	—
	$50,000 to $74,999	17.7	12.2	19.1	—
	$75,000 or more	32.6	16.0	36.7	—

^a^Weighted percentage. Missing value was excluded.

^b^Rao-Scott chi-square test. Missing value was excluded.

^c^Single included divorced, widowed, separated, single, or never been married.

^d^All monetary values presented in USD.

**Table 2:** Characteristics associated with electronic health information communication.

Characteristic		Total^a^ (n=3185)	Any disability^a^ (n=796)	No disability^a^ (n=2274)	*P* value^b^
**Regular provider, %**					
	Yes	63.6	71.2	61.7	.003
	No	36.4	28.8	38.3	—
**Electronic medical record use by health care provider, %**					
	Yes	87.7	89.0	87.2	.47
	No	12.3	11.0	12.8	—
**Mobile device, %**					
	Have one or more than 1 electronic mobile device (eg, mobile phone)	91.7	82.5	94.1	<.001
	No mobile device	8.3	17.5	5.9	—
**Importance of patient accessing medical information electronically, %**					
	Very important	64.9	58.2	66.5	.009
	Somewhat/not at all important	35.1	41.8	33.5	—
Willingness to exchange medical information with provider, mean (SD)		2.57 (0.03)	2.39 (0.07)	2.62 (0.03)	<.001

^a^Weighted percentage. Missing value was excluded.

^b^Rao-Scott chi-square test for categorical variables and *t* test for continuous variables. Missing value was excluded.

**Table 3:** Predictors of seeking information from health care provider and internet as health information source.

Predictors		Internet								Provider							
		As actual source^a,b^			As hypothetical source^b,c^				As actual source^a,b^					As hypothetical source^b,c^			
		AOR^d^	95% CI	*P*		AOR^d^	95% CI	*P*			AOR^d^	95% CI	*P*		AOR^d^	95% CI	*P*
Disability (ref: no disability)		0.65	0.43-0.98	.048		0.66	0.41-1.06	—			1.24	0.76-2.04	—		1.25	0.77-2.03	—
Aged 65 years or older (ref: younger than 65 years)		0.30	0.21-0.45	<.001		0.45	0.30-0.68	<.001			2.07	1.36-3.14	<.001		1.88	1.28-2.75	<.001
**High trust (ref: low trust) with information from**																	
	Internet	3.62	2.07-6.33	<.001		2.53	1.63-3.92	<.001			0.38	0.19-0.78	<.001		0.47	0.32-0.69	<.001
	Provider	1.23	0.82-1.84	—		0.54	0.40-0.71	.001			1.50	0.93-2.40	—		2.26	1.71-2.99	<.001
	Family and friends	0.38	0.20-0.70	.003		0.74	0.40-1.36	—			1.41	0.62-3.21	—		0.88	0.49-1.57	—
High health literacy (ref: low health literacy)		0.71	0.48-1.06	—		1.31	0.94-1.83	—			1.47	0.87-2.49	—		0.84	0.59-1.18	—

^a^Study outcome actual source (provider and internet) for health information was measured by HINTS A2.

^b^*c*-statistic was used to evaluate the goodness of fit of the models. We reported the range of c-statistics for each set of the models using 10 imputed datasets: (1) internet as the actual source: 0.788-0.804; (2) internet as the hypothetical source: 0.729-0.743; (3) provider as the actual source: 0.721-0.751; (4) provider as the hypothetical source: 0.685-0.696.

^c^Study outcome hypothetical source (provider and internet) for health information was measured by HINTS A8.

^d^AOR (adjusted odds ratios): logistic regression model adjusted for gender, age group, marital status, education, perception on the importance of patient accessing personal health record, income, health insurance, having a regular provider, owning any mobile device, general health status, and electronic medical record system.

**Table 4:** Association between disability types and use of health information.

Type of disability	Seeking information from internet as actual source			Seeking information from provider as actual source			Exchanging health information with provider		
	AOR^a^	95% CI	*P* value	AOR^a^	95% CI	*P* value	AOR^a^	95% CI	*P* value
Hearing	0.58	0.32-1.06	—	1.75	0.78-3.90	—	1.09	0.76-1.56	—
Vision	0.27	0.11-0.65	.003	1.62	0.51-5.10	—	1.55	0.99-2.43	—
Cognition	0.69	0.37-1.27	—	1.21	0.58-2.52	—	1.15	0.89-1.48	—
Mobility	0.51	0.30-0.88	.02	1.60	0.89-2.85	—	1.16	0.87-1.53	—
Self-care	0.46	0.17-1.27	—	2.50	0.80-7.86	—	1.27	0.77-2.10	—
Independent living	0.55	0.25-1.25	—	1.67	0.67-4.14	—	1.47	0.94-2.29	—

^a^AOR (adjusted odds ratios): logistic regression model for each disability subgroup, with reference group as participants without any disability, adjusted for gender, age group, marital status, education, health literacy, trust toward information sources (provider, family and friends, internet, media, government health agencies, charitable or religious organizations), perception on the importance of patient accessing personal health record, income, health insurance, having a regular provider, owning any mobile device, general health status, and electronic medical record system.

## Discussion

### Principal Findings

This study analyzed nationally representative survey data on the association between disability and the use of the internet for health information and the use of IT to exchange information with a medical provider. Findings suggested a potential digital gap existed among adults with disabilities in using the internet to obtain health information, in particular for those with vision and mobility problems, but not in using IT to exchange medical information with a provider.

We also found associations between trust factors and health information obtaining behaviors. Those with a higher level of trust toward the internet for health information were significantly more likely to refer to the internet for health information compared to their counterparts in both their recent search and hypothetical scenarios. In addition, our study suggests that those who were more willing to exchange medical information via IT with provider were significantly more likely to do so compared to their counterparts. There was a significant difference in willingness to exchange medical information between those with and without disability, but the clinical significance of this difference is unclear. However, these results were consistent with previous research, indicating that psychosocial factors such as trust toward certain sources of health information and willingness to share medical information with a provider were significant predictors for actual use [37]. The trust factors represent potential areas for intervention to bridge the digital gap. Trust is a complex attitude that may encompass prior experiences with their providers and health care system. Improving the validity of websites through accredited and certified professional organizations along with enhancements to the comprehensibility of content might contribute to building trust toward health information online for those with disabilities [37].

In this study, we identified that disability conditions, specifically those affecting vision and mobility, were associated with decreased internet use to obtain health information. Implementing user interface incorporating features to enlarge font sizes and handy navigating tools may improve readability, build trust, and eventually improve the use of internet for health care services among adults with disabilities [37-39]. This also applies to the willingness to exchange medical information with a health care provider via IT. Having a reliable and user-friendly platform to exchange such information may help increase patients’ willingness to engage in exchanging health information with a provider. Future studies should evaluate the specific needs of those with disabilities and incorporate such needs in the design of websites in order to allow those with disabilities to obtain and exchange the relevant health information as conveniently as possible.

Another noticeable finding was that there were more individuals who reported they had used the internet to obtain health information in recent search than in hypothetical scenarios. We conducted subsequent analysis to examine the association between disability status and this observed discrepancy through logistic regression adjusting for the same covariates described earlier in this paper. Disability status was not significantly associated with this discrepancy, nor were the other covariates. The survey question for hypothetical scenario emphasized the strong need for health information, whereas such need was not specified in the actual scenario, which may contribute to the difference in measuring the 2 distinct outcomes. But more importantly, this observed discrepancy may reflect known barriers for patients to access a health care provider outside of the scope of this study [40,41]. Thus, people turned to the more readily available internet.

### Limitations

There are several limitations with this study. Because this is a retrospective cross-sectional study, a causal relationship cannot be established between disability status and study outcomes. Due to the nature of the secondary data, not all variables of interest were available for the analyses. For example, factors such as social support that could substantially impact the individual’s activity and societal participation were not available. Also, although the survey was designed to capture nationally representative data, certain selection bias and nonresponse bias cannot be ruled out. The overall survey response rate was relatively poor (35.2%), and the majority of the survey respondents completed the survey in English (94.7%) [30]. In addition, misclassifications of information sources due to the recall bias could potentially exist. Last, disability status was self-reported, which could affect the validity of the measure. There is limited information about whether respondents, especially those with disability, had used proxy respondents to help complete the survey. The weighted percentages of all 6 domains of disability were comparatively lower than the national estimates [1], suggesting a potential underrepresentation of the disability community.

### Conclusion

We found a potential digital gap among US adults with disabilities in terms of their recent use of the internet for health information but not in their IT use to exchange medical information with a provider. Also, we found trust toward health information source and willingness to exchange medical information with a health care provider were associated with the health information seeking behavior. This study contributes to the understanding of the disparities in accessing health information today among US adults with disability using a nationally representative survey. Future work should evaluate the potential for enhancing exchanges between those with disabilities and their providers through referrals to well-established and high-quality websites. Additionally, studies should focus on developing specific tools to improve access and develop trust with these sources to bridge the digital gap among those with disabilities.

## Appendix

Multimedia Appendix 1 Distribution of disabilities.

## Abbreviations

AOR: adjusted odds ratio
HINTS: Health Information National Trends Survey
IT: information technology
OR: odds ratio
