# Supplementary material for: Understanding the Digital Gap Among US Adults With Disability: Cross-Sectional Analysis of the Health Information National Trends Survey 2013
Source: JMIR Rehabil Assist Technol. 2018 Mar 16;5(1):e3. doi: 10.2196/rehab.8783 (PMC5878361; doi:10.2196/rehab.8783)
Supplement: Multimedia Appendix 1 [file rehab_v5i1e3_app1.pdf]

Table 1. Distribution of disabilities.

Type of disability	Total sample with any disability (N=796)	
	N[a]	%[b]
Hearing	242	29.9
Vision	157	18.3
Cognition	310	49.1
Mobility	459	48.6
Self-care	124	12.5
Independent living	217	23.4

Note: Disability subgroups are not mutually exclusive.

[a] Unweighted frequency.

[b] Weighted percentage in total sample or by age group. Missing excluded.
